# Conservation of Resources, Psychological Distress, and Resilience During the COVID-19 Pandemic

**DOI:** 10.3389/ijph.2022.1604567

**Published:** 2022-08-31

**Authors:** Hadas Egozi Farkash, Mooli Lahad, Stevan E. Hobfoll, Dima Leykin, Limor Aharonson-Daniel

**Affiliations:** ^1^ School of Public Health, Faculty of Health Sciences, Ben-Gurion University of the Negev, Beer-Sheva, Israel; ^2^ Department of Psychology, Tel-Hai College, Tel-Hai, Israel; ^3^ The Community Stress Prevention Centre (CSPC), Kiryat-Shmona, Israel; ^4^ STress Anxiety and Resilience Consultants-STAR, Salt Lake City, UT, United States; ^5^ PREPARED Center for Emergency Response Research, Ben-Gurion University of the Negev, Beer-Sheva, Israel

**Keywords:** loneliness, COVID-19, resilience, stress, Conservation of Resources theory, personal resilience, community resilience CCRAM, traumatic symptoms

## Abstract

**Objectives:** The Conservation of Resources (COR) theory suggests that stress results from threatened or actual loss of resources following significant life events. This study used COR theory as the framework to explore the reflection of loss of resources during the COVID-19 pandemic on psychological distress and resilience, in an adult Jewish Israeli population.

**Methods:** We examined the association between background variables, stress, loneliness, concern, COVID-19-related post traumatic symptoms (PTS), resilience factors and COR *via* an online survey among 2,000 adults during April 2020.

**Results:** Positive relationships were identified between resource loss and PTS (*r* = 0.66, *p* < 0.01), and between resource gain and resilience (*r* = 0.30, *p* < 0.01). Psychological variables were significantly associated with PTS and explained 62.7% of the variance, *F* (20, 1,413) = 118.58, *p* < 0.001.

**Conclusion:** Loss of resources, stress, loneliness and concern were found to be risk factors for distress and PTS, whereas resilience factors played a protective role. We thus recommend using the COR theory to explore COVID-19 effects elsewhere.

## Introduction

The socio-psychological consequences of the COVID-19 pandemic are widespread, possibly affecting mental health not only now but into the future [1]. Although the population of Israel is known for its resilience to security-related crises, it is not known how they would face an epidemic threat. With the growing availability of the vaccine, it is important to deepen our understanding of the psycho-social distress and coping of the population. Hopefully, such understanding will lead to wide-scale interventions to help restore psychological equilibrium as the world moves towards learning to live with COVID-19.

The COVID-19 pandemic could leave many people with psychological scars, such as depression [2] anxiety, stress, traumatic symptoms [3], and loneliness [4], which are common reactions to a health crisis. The pandemic raised the general anxiety levels [5]. Thus, uncertainties about the future, anxiety and fear may last even after COVID-19 is eradicated or contained. Loneliness as well, has been found to be a central risk factor for depression, anxiety, and comorbidity during the COVID-19 pandemic [4]. The widely adopted social and physical distancing, lockdowns, isolation and quarantine have not surprisingly resulted in severe psychiatric disorders [6], as did the emotional burden of loneliness [7]. However, mental health researchers suggest viewing the COVID-19 crisis from a broader trauma perspective [8]. Seen this way, infectious disease epidemics can be highly traumatic experiences for individuals and lead to post-traumatic stress disorder (PTSD) and chronic psychological distress, especially for those already vulnerable or who are most deeply impacted by the disease and its co-travellers [9].

The COVID-19 pandemic and its effects on people’s lives highlight the need to deepen our knowledge in the concepts of resilience and human resources and their impact on distress and coping. Personal Resilience is defined as a trajectory of healthy functioning after a highly adverse event [10]. Lahad [11] claims that some individuals have a unique resource repertoire that helps them deal with crises. Studies of personal resilience during the COVID-19 pandemic have shown a link between high personal resilience and decreased anxiety, distress, feelings of danger, depression and anxiety [12].

Community resilience is defined as “the community’s ability to withstand crises or disruptions” [13], and is another resource that may be supportive for individuals during a global pandemic, particularly when people were asked to remain in their physically close domain.

The Conservation of Resources theory (COR) [15] has often been adopted to study major and traumatic stress in crisis situations, and has been referenced as a framework for understanding stress in many studies. According to COR theory, people strive to retain, protect, and build resources and are threatened by the potential or actual loss of these valued resources. Resources may be material (e.g., money, housing), social (e.g., social support, status) or psychological (e.g., personal mastery, sense of autonomy) [15]. Loss of resources, or threat of such loss, is a crucial variable, predicting psychological distress, and will lead to investing more resources, making those already lacking in resources even more vulnerable to loss spirals [15]. Concrete primary-resource losses and secondary losses that occur later, were found to best predict psychological distress during major crisis [16].

COR theory also emphasizes resource gain, such that gain of important resources and associated positive emotions may increase in value in the face of loss. When resource loss occurs, the ability to gain resources becomes increasingly important, providing an emotional respite and the ability to achieve goals [17]. As such, following a crisis, resource loss is seen as more impactful than resource gain, which is seen to have more modest, even if important influence.

Despite its important contribution, the association between COR theory and the COVID-19 pandemic has been studied only to a limited extent [18–21]. The physical health, economic and socio-psychological consequences of COVID-19 inevitably result in multiple forms of loss of resources (e.g., loss of a loved one, health, financial stability, job, social connection and sense of security), which may increase the likelihood of developing traumatic symptoms and psychological distress [9].

Among the studies examining the impact of the COVID-19 pandemic on mental health, the impact of resource loss and gain (COR) has received little attention. To fill this gap, the present study examined COR and its association with personal and community resilience as well as psychological distress.

The study objectives are:

(1) To examine the association between COR and psychological distress related to COVID-19,
(2) To examine the association between COR and resilience during the COVID-19 pandemic.

Hypotheses:

Adjusted for demographic and psychological variables, resource loss will be associated with an increase in psychological distress, and resource gain will be associated with an increase in resilience factors.

## Methods

### Data Collection

Data were collected through an online survey distributed for 4 days in April 2020, to participants in Israel at the time of the pandemic. The survey was sent through Midgam Project Web Panel, an Israeli company specializing in internet research *via* an online panel (www.midgampanel.com), where panelists are paid to participate in periodic surveys. Participants are sampled using a stratified quota sampling. The study was pre-approved by the Institutional Review Board (IRB) of the Faculty of Health Sciences at Ben-Gurion University of the Negev. An introduction at the beginning of the survey described the study’s objectives and specified that completing the questionnaire was voluntary and could be terminated at any time. According to the checklist for reporting results of internet [22], it was possible to change the answers before submitting the questionnaire. The questionnaires were anonymous to the research team, and each user could participate only once using a unique identifier.

### Participants

Of the 2,302 panelists who responded to the survey, 2,000 participants provided complete responses (86.9%). Participants were Jewish adults (aged 17–74 years), residents of Israel, representing the adult Jewish population in Israel in the examined age range.

### Measures

#### Resource Loss and Gain

A modified version of the Conservation of Resources Evaluation Questionnaire (COR-E) [23] was used, with 7 items representing perceived loss of resources and 7 items representing perceived gain of resources relevant to the pandemic. Loss of resources (such as loss of meaning and purpose, financial stability, etc.) was rated on a 5-point Likert scale (0—I did not lose at all, 4—I lost very much). The gain items (such as meaning and purpose, closeness to family and friends) were rated on a 5-point Likert scale (0—I did not gain at all, 4—I gained very much). Internal reliability for resource loss (*α* = 0.87) and for resource gain (*α* = 0.84) in study population were good.

#### COVID-19-Related Traumatic Symptoms

The items used to measure traumatic symptoms related to COVID-19 were taken from three validated questionnaires: CAPS-5 [24], PDS [25], and Patient Health Questionnaire - PHQ-9 [26]. The 18 items (e.g., “I have troubling thoughts about events related to Corona”) examined the existence of continuous traumatic symptoms associated with COVID-19 according to *DSM-5* criteria [27]. The responses were rated on a four-point Likert scale (0—not at all, 3—very much). Internal reliability was high (*α* = 0.90).

#### COVID-19 Related Concern

The 7-item measurement tool was the situational anxiety questionnaire [28], based on the Spielberger [29] State-Trait Anxiety Inventory (STAI). The 7 items (e.g., “I am worried about the spread of the coronavirus”) were rated on a 5-point Likert scale (0—not at all, 4—very much). Internal reliability was high (*α* = 0.88).

#### Loneliness

The revised UCLA Loneliness Scale [30] was used to measure loneliness. Of the 20 items in the scale, three were used in the current study (e.g., “How many times in the last month have you felt: that you have no one to turn to”), were rated on a 5-point Likert scale (0—never, 4—always). Internal reliability was good (*α* = 0.80).

#### Personal Resilience

Personal resilience was measured by the 10-item Conor Davidson scale (CD-RISC10, [31]. The statements (e.g., “I can deal with unpleasant feelings”) were rated on a 5-point Likert scale (0—not at all, 4—largely correct). Internal reliability in the present study was high (α = 0.88).

#### Community Resilience

A modified version of the Conjoint Community Resiliency Assessment Measure (CCRAM-10 [14]; was used. The 6 items (e.g., “The community in which I live in is functioning properly”) were rated on a 5-point Likert scale (0—strongly disagree, 4—strongly agree). Internal reliability was high (*α* = 0.89).

#### Background Information

Participants were asked about their background. Questions referred to gender, marital status, dependents, housing, education, religiosity, birthplace, health status, income, history of quarantine, and volunteer activity.

### Data Analysis

Data were analyzed using Statistical Package for the Social Sciences (SPSS) version 25 and JASP version 13. Only completed questionnaires were analyzed. Mean and Standard Deviation indices were calculated.

Cronbach’s alpha was used to examine each component of the survey instrument -COR-E, COVID-19-related traumatic symptoms, COVID-19 related concern, loneliness, personal resilience and community resilience reliability. Pearson correlation coefficients were calculated and used to examine the association between resource loss, resource gain, resilience factors, psychological distress factors and background variables. Additional Pearson tests were performed to examine the association between traumatic symptoms and resource loss items, the association between traumatic symptoms and concern items, and between personal resilience and resource gain statements. Subsequently, hierarchical linear regression modeling was performed to predict COVID-19-related traumatic symptoms adjusted to demographic and psychological variables. In the first step, the demographic variables were brought under control, and in the second step, the psychological and other variables were introduced. *p*-values are reported at a significance level of *p* = 0.05 and *p* = 0.01.

## Results

### Study Variables

As presented in Table 1, the mean age of the participants was 42.25 years (*SD* = 15.73, range 17–74 years). About half of the respondents were men (*n* = 995, 49.75%). Most participants (*n* = 1,055, 59%) were married or cohabiting. The majority of the participants reported attaining post-secondary (*n* = 564, 28.2%) or academic education (*n* = 885, 44.25%). About 75% (*n* = 1,489) of the sample reported being generally healthy. As related to the pandemic—14.9% (*n =* 299) were quarantined at the time of measurement; only 0.4% (*n* = 8) of the participants were tested positive to COVID-19 by the time of the survey (early April 2020).

**TABLE 1:** Demographic characteristics of respondents (*N* = 2000). Conservation of resources during the COVID-19 pandemic, Israel, 2020.

Variables	*N*	%
Gender		
Female	1,004	50.20
Male	995	49.75
Marital status		
Married/cohabiting	1,055	59.05
Single	915	40.95
Age		
15–24	305	15.25
25–34	436	21.80
35–44	397	19.85
45–54	320	16.00
55–64	309	15.45
65+	233	11.65
Children under 16		
0	884	44.20
1	315	15.70
2	227	11.30
3	130	6.50
4+	69	3.30
Number of people at home		
0	86	4.30
1	272	13.60
2	478	23.90
3	343	17.10
4	330	16.50
5	286	14.30
6+	203	10.00
Dependents		
Special needs	72	3.60
Dependent child	519	25.95
Dependent adult	197	9.85
Over 70	175	8.75
House type		
Apartment	1,393	69.65
House	572	28.60
Garden/view in the house	1,604	80.20
Education		
University/college	885	44.25
Post-secondary education	564	28.20
High school	494	24.70
Elementary school	21	1.05
Religiosity		
Religious	793	29.16
Secular	1,207	60.35
Birthplace		
Israel	1,613	80.65
Not in Israel	387	19.35
Health status		
Healthy	1,489	74.45
Chronic illness	477	23.85
Income		
Above average	296	14.80
Average	544	27.20
Below average	944	47.20
Quarantined	299	14.95
SARS-CoV-2 positive	8	0.40
Volunteering in a routine	471	23.50
Volunteering during COVID-19	309	15.40

Table 2 presents descriptive statistics and Pearson correlations between study variables. As seen in Table 2, resource gain’s mean score (M = 1.71, SD = 1.01) was higher than resource’s loss mean score (M = 1.03, SD = 0.86).

**TABLE 2:** Means, standard deviations, and Pearson correlations between study variables. Conservation of resources during the COVID-19 pandemic, Israel, 2020.

	Mean	SD	1	2	3	4	5	6	7	Range
1. COVID-19-related concern	1.94	1.90								0–4
2. Stress	1.49	0.94	0.66**							0–4
3. Loneliness	1.36	0.96	0.29**	0.49**						0–4
4. Personal resilience	3.09	0.67	−0.18**	−0.31**	−0.29**					0–4
5. Resources loss	1.03	0.86	0.34**	0.52**	0.50**	−0.37**				0–4
6. Resources gain	1.71	1.01	0.07**	−0.07**	−0.14**	0.21**	−0.08**			0–4
7. Community resilience	2.46	0.94	−0.08**	−0.16**	−0.21**	0.23**	−0.25**	0.30**		0–4
8. COVID-19 related traumatic symptoms	0.72	0.53	0.47**	0.69**	0.56**	−0.44**	0.66**	−0.09**	−0.22**	0–3

*p < 0.05, **p < 0.01.

**FIGURE 1:**
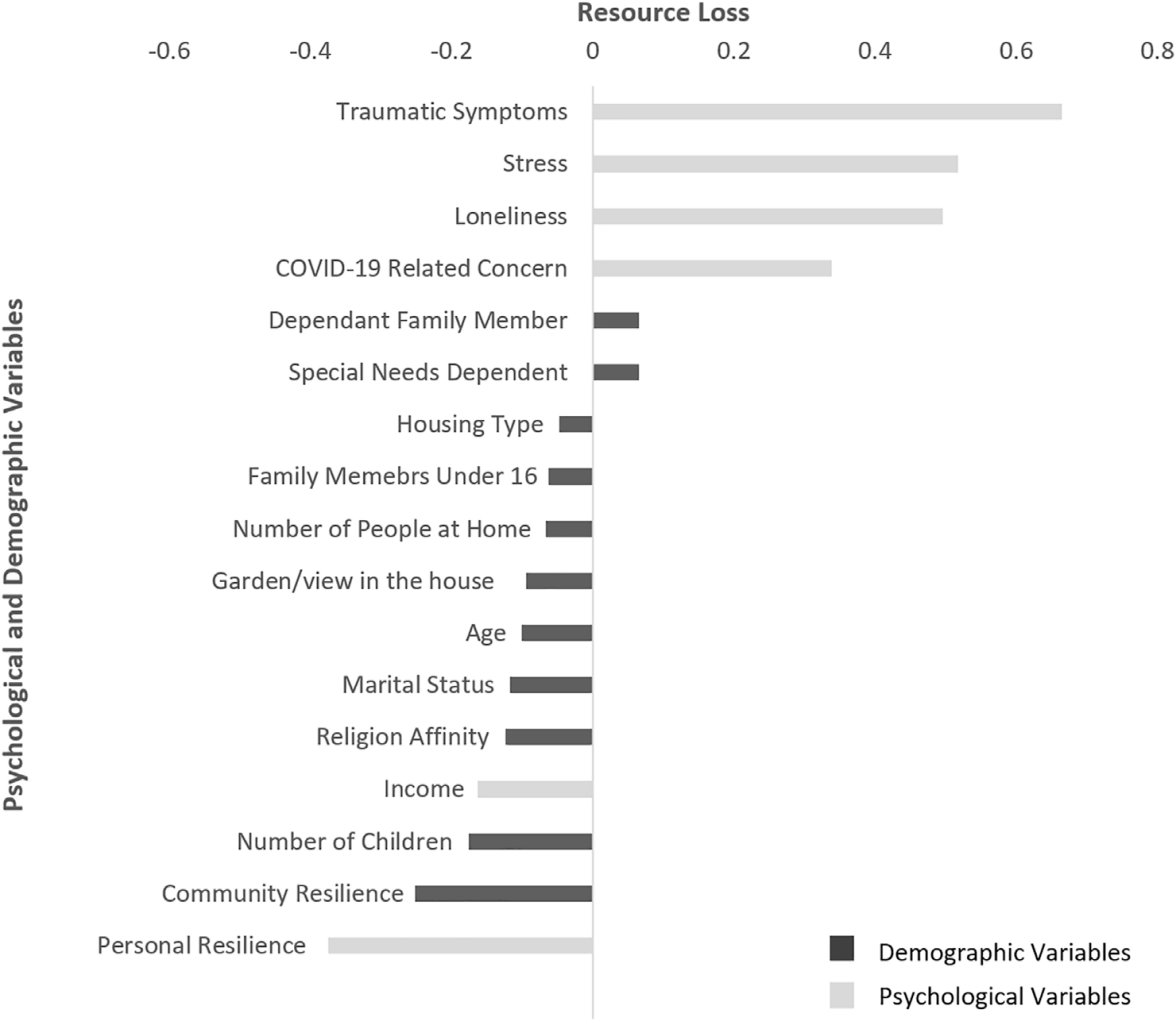
Correlations between demographic and psychological variables and resource loss. Conservation of resources, psychological distress and resilience during the COVID-19 pandemic, Israel, 2020.

Figure 1, focuses on the relationships between resource loss and study variables and demonstrates the large positive correlation between resource loss, COVID-19-related traumatic symptoms (*r* = 0.66, *p* < 0.01); stress (*r* = 0.52, *p* < 0.01); loneliness (*r* = 0.50, *p* < 0.01); COVID-19 related concern (*r* = 0.33, *p* < 0.01). Negative associations were found between resource loss and personal resilience (*r* = −0.37, *p* < 0.01) and between resource loss and community resilience (*r* = −0.25, *p* < 0.01).

**FIGURE 2:**
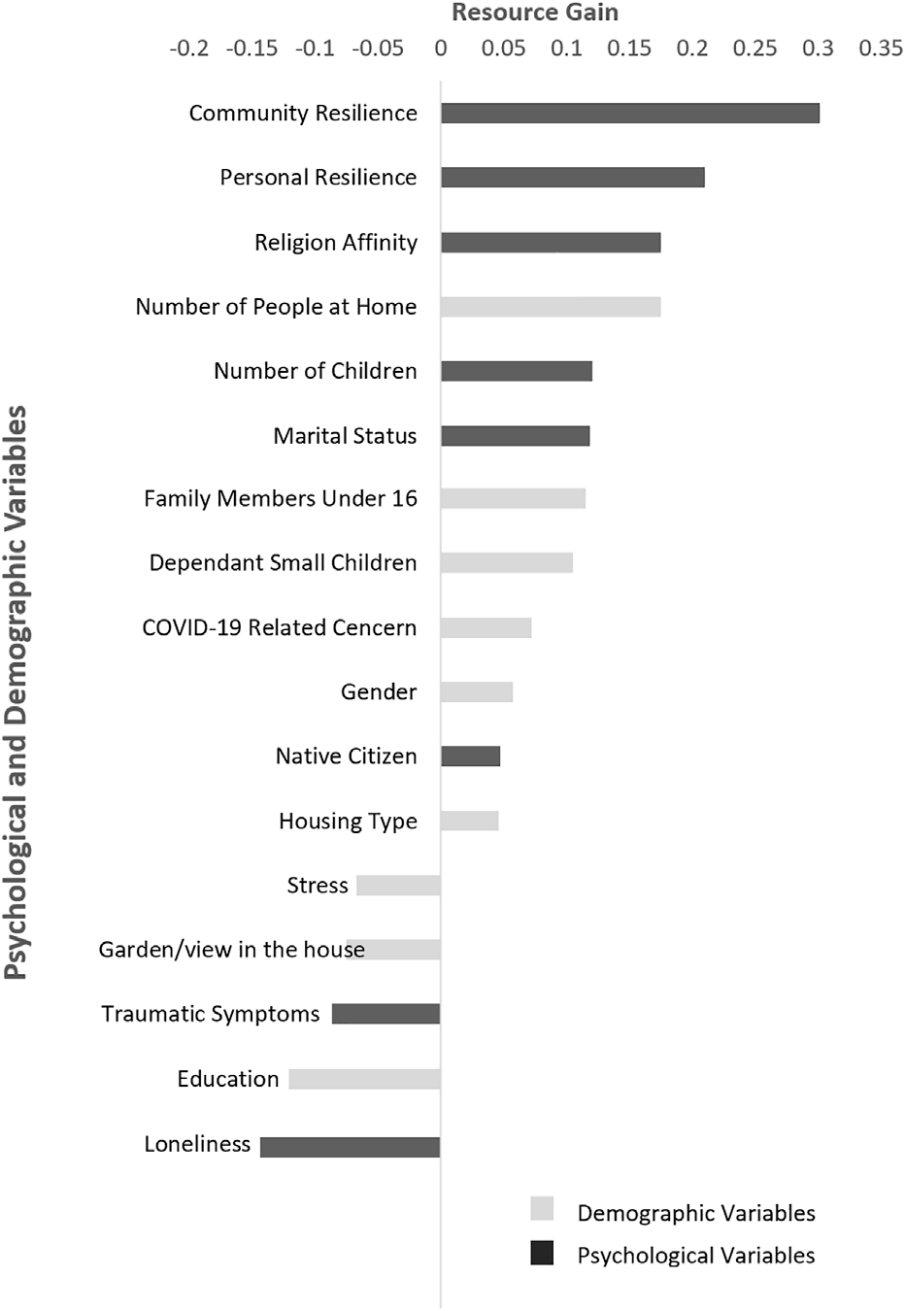
Correlations between demographic and psychological variables and resource gain. Conservation of resources psychological distress and resilience during the COVID-19 pandemic. Israel, 2020.

Figure 2 portrays the relationships between resources gain and study variables. Positive correlations were noted between resource gain and community resilience (*r* = 0.30, *p* < 0.01) as well as between resource gain and personal resilience (*r* = 0.30, *p* < 0.01).

### Resource Gain and Loss Items and Their Relationships to Resilience Variables

**FIGURE 3:**
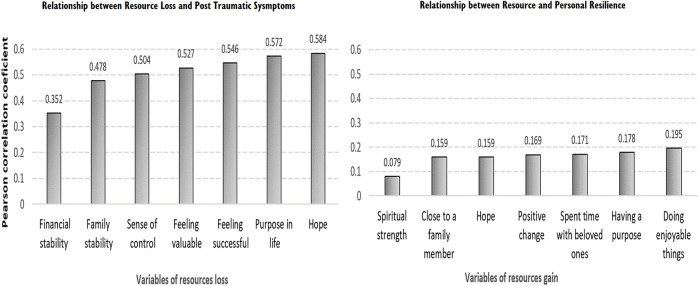
Relationship between resource loss and PTS and personal resilience. conservation of resources psychological distress and resilience during the COVID-19 pandemic, Israel, 2020.

To examine the relationships between resource gain and personal resilience, a Pearson correlation test was performed. Figure 3 shows the relationships between resource gain items and personal resilience. The strongest correlation was found between personal resilience and “doing enjoyable or important things” (*r* = 0.18, *p* < 0.01).

To examine the links between traumatic stress symptoms and resource loss items, additional Pearson tests were performed. Figure 3 further demonstrates that loss of hope had the highest association with the possibility of COVID-19 related traumatic symptoms (*r* = 0.58, *p* < 0.01), followed by a loss of a sense of meaning or purpose in life (*r* = 0.57, *p* < 0.01), and a loss of a sense of success (*r* = 0.54, *p* < 0.01), and value (*r* = 0.52, *p* < 0.01). Financial stability had the lowest association with COVID-19-related traumatic symptoms (*r* = 0.35, *p* < 0.01).

Significant, yet weak, correlations were found between health status and most of the study variables. Specifically, healthy individuals were found to have less resource loss (*r* = −0.07, *p* < 0.01), more resource gain (*r* = 0.09, *p* < 0.01), less COVID-19-related traumatic symptoms (*r* = −0.11, *p* < 0.01), less COVID-19 related concern (*r* = −0.09, *p* < 0.01), less loneliness (*r* = −0.06, *p* < 0.01), higher personal resilience (*r* = 0.05, *p* < 0.05) and higher community resilience (*r* = 0.05, *p* < 0.05), than individuals whose health status was poor.

### Traumatic Symptoms Related to COVID-19 Situation

To examine which of the demographic and psychological variables is associated with COVID-19-related traumatic symptoms, a hierarchical linear regression was performed. In the first step, the demographic variables were entered, and in the second step, the psychological and other variables were added.

Background variables explained 6.8% of the variance in COVID-19-related traumatic symptoms, *F* (12, 1,421) = 9.68, *p* < 0.001. Specifically, having a dependent family member or dependent person with special needs (*β* = 0.09, *p* < 0.01), religiosity (*β* = -0.10, *p* < 0.01), low income (*β* = −0.07, *p* < 0.01), and being female (*β* = 0.10, *p* < 0.01) were related to more severe traumatic symptoms (see Table 3).

**Table 3:** Hierarchical Regression Coefficients associated with COVID-19-related Traumatic Symptoms according to Demographic and Psychological Variables (*N* = 1,425). Conservation of Resources Psychological Distress and Resilience during the COVID-19 pandemic, Israel, 2020.

Variable	Model 1			Model 2		
	*B*	(*β*)	S.E.B.	*B*	(*β*)	S.E.B.
Step 1						
Age	−0.00	−0.05	0.00	−0.00	−0.03	0.00
No. of household members under 16	0.00	−0.01	0.01	0.00	0.00	0.01
Dependent family member	0.17	0.09**	0.04	0.06	0.03*	0.03
Dependent person with special needs	0.25	0.09**	0.07	0.08	0.03	0.04
Housing type	−0.06	−0.06*	0.03	−0.00	−0.00	0.02
Garden/view in the house	0.05	0.03	0.03	−0.03	−0.02	0.02
Religiosity	−0.05	−0.10**	0.01	−0.01	−0.02	0.01
Living alone	−0.01	0.03	0.01	−0.01	−0.03	0.00
Marital status	−0.03	−0.02	0.03	−0.02	−0.02	0.02
Income	−0.03	−0.07**	0.01	0.00	0.00	0.00
Gender (female)	0.10	0.10**	0.02	0.03	0.32	0.01
No. of children	−0.02	−0.07	0.01	0.01	0.04	0.00
Step 2						
COVID-19-related concern				0.03	0.05*	0.01
Loneliness				0.08	0.15**	0.01
Stress				0.17	0.32**	0.01
Personal resilience				−0.12	−0.16**	0.01
Community resilience				−0.01	−0.02	0.01
Resource loss				0.20	0.33**	0.01
Resource gain				0.00	0.01	0.00
*R* ^2^		0.07**			0.62**	
Δ*R* ^2^		0.07**			0.55**	
*F*		9.68**			118.5**	

*p < 0.05 **p < 0.01.

Loneliness and stress, significantly contributed to COVID-19-related traumatic symptoms and explained 62.7% of the variance, *F* (20, 1,413) = 118.58, *p* < 0.001. Specifically, COVID-19-related stress (*β* = 0.32, *p* < 0.01) and resource loss (*β* = 0.33, *p* < 0.01) had the most significant individual contribution to COVID-19-related traumatic symptoms. Additional unique contributions were found with loneliness (*β* = 0.15, *p* < 0.01), low personal resilience (*β* = −0.16, *p* < 0.01). All of which were significantly associated with COVID-19- related traumatic symptoms.

## Discussion

The Conservation of Resources theory [15], emphasizing the association of the potential or actual loss or gain of resources with individuals’ well-being, was supported in the current study of the COVID-19 pandemic. We examined the associations between resources loss and gain, psychological distress, and resilience factors among Jewish adults in Israel at the early stage of COVID-19 when the country was in lockdown and vaccines were a far-off dream.

The COVID-19 pandemic can be highly stressful for all individuals. However, our study suggests that those who suffered more were females, people with lower-income, more secular affiliation, or a person with special needs dependent on them.

Our findings correspond with the psycho-social disturbances that were found in previous studies, particularly among those who had low socioeconomic status, job loss, or unemployment status following traumatic exposure [32]. In addition, they reflect reduced access to psycho-social and financial resources [33]. Resources like job and income are part of the individual’s self-identity, and their loss may reduce self-esteem, sense of security and sense of meaning in life. From this perspective, we can identify the COVID-19 pandemic as an existential crisis [34].

Level of religiosity was found to be negatively associated with traumatic symptoms. Religions can create a sense of meaning and trust in high powers [35]. Our study showed that spirituality seems to play a role in alleviating suffering and minimizing the consequences of social isolation [36].

During COVID-19, families are under multiple-stressors: trying to adapt to the uncertainty caused by the lockdowns, the fear for their members’ health, experiencing losses and the challenges of strengthening vital bonds, and overcoming difficulties [37]. Our results suggest that families with a dependent member, and of lower income, were more vulnerable than others. Preexisting vulnerabilities within families increase susceptibility to psycho-social disruptions during the COVID-19 pandemic [38]. In our study we found that large, religious, and upper-middle-class families have been able to gain more resources, comparing with low-class families with a dependent or disabled family member.

Being a female was found to be a risk factor for reporting more traumatic symptoms [39]. On the other hand, women reported higher resource gain scores than men. A possible hypothesis for these findings suggests that women’s greater tendency to ruminative thinking and their coping style, centered on emotion, facilitates both stress and growth [40]. Another possible explanation for women’s higher ability to feel stress and to perceive resource gain is that during the COVID-19 pandemic, the domestic challenges became the focus of coping, and there, traditionally, the woman has more tasks that were intensified during the lockdown situation (e.g. looking after kids and close relatives, cooking, etc.). Based on these mixed findings we would like to stress that it reflects the first wave of the epidemic, and it is still unclear how women will be affected by the epidemic over time.

Our first hypothesis, that a strong association will be found between resource loss and psychological distress levels, was confirmed by the current study. Furthermore it indicated that the psychological variables, especially loss of valued resources (such as hope, sense of meaning or purpose in life), had the most significant contribution as a risk factor for COVID-19-related traumatic symptoms. Additionally, significant positive correlations were found between resource loss and psychological distress such as traumatic symptoms related to COVID-19: loneliness, stress, and COVID-19-related concerns) about the spread of the coronavirus, changes in daily routine, personal and relative’s health and threaten on life).

Our findings thus support the Conservation of Resources theory (COR) which emphasizes that loss of resources can challenge the ability of individuals to cope with and recover from traumatic situations [15]. In a stressful global pandemic, multiple types of loss threatened or caused a depletion of people’s resources, and was associated with an increase in stress levels. Psycho-social resource loss can be seen as a spiral of deficit the longer the crisis persists and affects other areas of life [41].

Our findings suggest that resource loss was highly associated with loneliness, a crucial factor in the COVID-19 epidemic [42]. Of all the losses examined in the current study, loss of hope had the highest association with COVID-19-related traumatic symptoms. Hope as a resilience factor might reduce psychological distress [43], and hopeful people are better able to respond to challenging situations.

Previous studies have shown a chain mediation model using other variables, showing that health information and the perceived impact of the pandemic were mediators that contributed to mental health [44], and other variables (such as coping strategies) have mediation effect on our research variables [45]. Further studies will focus on the identification the moderating and mediating variables of our study’s variables over time.

Our second hypothesis focused on the association between resource gain and resilience factors, was also confirmed. A significant positive correlation was found between resource gain, community resilience, and personal resilience. As predicted by the COR theory, resource gain’s influence was substantively less powerful than that of resource loss [23]. However, our findings suggest that overall, the Israeli Jewish population exhibited a greater resource gain than resource loss in the first wave of the COVID-19.

The correlation found between resource gain and resilience factors highlights the importance of resource gain aspect when dealing with crises. People who had higher resource gain reported doing more enjoyable or valuable things and having a sense of purpose in life. These results are supported by the post-traumatic growth concept (PTG) [46], which focuses on positivity when individuals experience adversity and contains a transformative potential, whereby a person grows in appreciation of life, envisioning new possibilities, personal strength, and spiritual understanding [46].

Personal and community resilience were found to be the best protective factors for decreased psychological distress**.** Our findings support other studies that presented the crucial role of resilience factors, especially of personal resilience, on mental health when coping with COVID-19 threats [47].

Resilience refers to the extent to which people could manage to create and sustain resource gains, it acted as a vital protector that enables the ability to “bounce forward” despite the pandemic challenges and maintain functioning.

As data for this study were collected at the early stages of the pandemic, the rate of illness and death at the time was still low compared to later stages. Future work should follow these measures repeatedly and address the association between aspects such as COVID-19 morbidity and mortality and changing socio-economic status on the variables measured in this study.

### Limitations

The use of an online approach to data collection through the MIDGAM web panel limited us to the population in their database which represents the computer literate Jewish population only. Nevertheless, the vast majority of Israels’ population uses the Internet (84% in January 2020, 88.0% in January 2021) [48]. The MIDGAM web panel [49] has access to hundreds of thousands of Israelis who are interested in partaking in online studies, usually for monetary reimbursement and provides a statistically representative sample of the adult Jewish population in Israel, so in terms of the population of this study, it is deemed sufficient. Furthermore, at the time of the study, when people were confined home, it was the best possible way, as only 53% of the households have a landline [50].

Previous studies during the COVID-19 pandemic highlighted this susceptibility of selection bias, claiming that large sample sizes, not necessarily compensate for that bias and might even exacerbate it [51]. Our findings thus have the potential to not fully represent the general population and to limit the interpretations of the findings to the population described above (higher educational level for example). Despite that, based on previous studies using this panel company [5], we feel that it is safe to assume that the sample offers insights which are representative of the adult Jewish population in Israel. Beyond that, it is important to remember that we did not try to characterize the population or to infer about it as a population but rather examined a human psycho-social experience and the relationship between people’s available resources, their psychological distress and resilience. Therefore, the meaning of this potential bias could be marginal. Nevertheless, it is necessary to test our findings on diverse samples in order to confirm the study’s validity on other populations.

### Conclusion and Recommendations

The COR theoretical framework offers a prism for understanding people’s coping with traumatic experiences, and offers important insights that can serve to plan support for individuals and communities in the context of COVID-19. The study supports the need for handling the COVID-19 mental health consequences as part of the national systemic response. Interventions should focus on both the physical and environmental levels (money, agile working conditions etc.) and people’s internal resources (such as creating new meanings in life) in order to reduce psychological negative effects. Precisely at time of crises, resources are inaccessible and are critical to recovery. The ongoing loss should be ceased in the early stages, before the acceleration of resource loss and negative effect of loss spirals. Acquiring new psychological resources may be a tool in cultivating mental well-being. This awareness-raising may be part of the role of policymakers, education and health systems leaders and it should be conveyed by the media as well.

Our study shows that it is important to develop resilience skills, cultivate hope and create mental-health interventions to vulnerable populations that have experienced a loss of resources due to this pandemic and those who suffer from loneliness. Actually, the findings that reflect the constant vulnerability of part of the population to distress and their limited access to resources, should raise concern, as it reflects the need for policy of caring for populations which are vulnerable to crises. Local leaders and digital communities’ leaders should take a role in strengthening community resilience in order to support mental well-being. The long-term social and psychological effects of COVID-19 are only starting to be known, yet based on the current research and other studies, and given the critical protective role of resilience, we recommend examining the concept of family resilience in the face of social challenges during a global epidemic.
